# Fast Online Optimization for Terrain-Blind Bipedal Robot Walking With a Decoupled Actuated SLIP Model

**DOI:** 10.3389/frobt.2022.812258

**Published:** 2022-02-18

**Authors:** Ke Wang, Hengyi Fei, Petar Kormushev

**Affiliations:** ^1^ Robot Intelligence Lab, Dyson School of Design Engineering, Imperial College London, London, United Kingdom; ^2^ Department of Electrical and Electronic Engineering, Imperial College London, London, United Kingdom

**Keywords:** bipedal robot, footstep planning, uneven terrain walking, optimization, model predictive control

## Abstract

We present an online optimization algorithm which enables bipedal robots to blindly walk over various kinds of uneven terrains while resisting pushes. The proposed optimization algorithm performs high-level motion planning of footstep locations and center-of-mass height variations using the decoupled actuated spring-loaded inverted pendulum (aSLIP) model. The decoupled aSLIP model simplifies the original aSLIP with linear inverted pendulum (LIP) dynamics in horizontal states and spring dynamics in the vertical state. The motion planning can be formulated as a discrete-time model predictive control (MPC) problem and solved at a frequency of 1 kHz. The output of the motion planner is fed into an inverse-dynamics–based whole body controller for execution on the robot. A key result of this controller is that the feet of the robot are compliant, which further extends the robot’s ability to be robust to unobserved terrain variations. We evaluate our method in simulation with the bipedal robot SLIDER. The results show that the robot can blindly walk over various uneven terrains including slopes, wave fields, and stairs. It can also resist pushes of up to 40 N for a duration of 0.1 s while walking on uneven terrains.

## 1 Introduction

To make bipedal robots really suitable for many applications, it is important that they can go out of the lab and walk in the complex real world environment. Real-world environments contain various kinds of uneven terrains: slopes, stairs, and hills. Most existing controllers that allow a bipedal robot to walk over uneven terrains require predefined footstep locations or exact information about the terrain height variations (Mordatch et al., 2010; Englsberger et al., 2015; Liu et al., 2015). However, even with most advanced sensors, there are some uncertainties on the perception of the terrain. In contrast, humans can easily walk on uneven terrains, such as outdoor environments, and without extra thought or careful planning. Therefore, it is important to have a reactive controller that is robust to unobserved uneven terrain variations.

The spring-loaded inverted pendulum (SLIP) model has become a popular model for walking and running in the legged robotic research (Raibert, 1986). Despite its simplicity, it has been proven to capture essential dynamics properties of walking and running (Geyer et al., 2006). The standard setting of a SLIP model is energy-conservative: it assumes there is no energy loss at impact. Though this assumption simplifies the control analysis, it does not resemble the reality. There is energy loss on physical systems, and robots (Ahmadi and Buehler, 2006; Robotics, 2017) designed to approximate SLIP dynamics have added actuation to compensate for the energy dissipation. As a result, the actuated spring-loaded inverted pendulum (aSLIP) model (Ernst et al., 2010) is proposed for a better approximation of the real robot dynamics. The aSLIP model has been successfully used to design controllers not only for SLIP-like robots (Apgar et al., 2018; Xiong and Ames, 2018; Green et al., 2020) but also as a template model for humanoid robots on uneven terrain walking (Liu et al., 2016).

An important step in making a controller reactive is to achieve real-time–constrained optimization. However, due to nonlinear dynamics that arises from the 3D aSLIP model, fast optimization is difficult. Liu et al., (2016) used a gait synthesized from a library of gaits acquired from off-line optimization, but this requires a large computation load off-line and cannot cover all possible situations Apgar et al., (2018) decoupled a 3D aSLIP model to facilitate fast computation, but the continuous dynamics of the decoupled aSLIP model is still nonlinear, and there is no theoretical guarantee of fast convergence. Xin et al., (2019) used the simpler linear inverted pendulum (LIP) model to design a reactive controller for flat ground walking, but this model cannot be applied to walking on uneven terrains.

Our article proposes a reactive controller that enables robots to blindly walk over uneven terrains by optimizing horizontal footstep locations and center-of-mass (CoM) height online. “Terrain-blind” means the robot is not provided with an environment map that specifies terrain height, but the robot can still get access to its own state through state estimation, for example, yhe CoM height. Under the assumption that the angle the modeled inverted pendulum makes with the vertical is relatively small, we can decouple the 3D aSLIP model into a 1D-actuated spring model responsible for *z* direction and 2D LIP model responsible for *x* and *y* directions. The dynamics of all three dimensions can be written as linear equations in a discrete-time state space manner. We formulate the online step planner as a discrete-time model predictive control (MPC) problem and solve it by quardratic programming (QP). To facilitate fast computation, the spring length is constrained to change linearly. As a result, we get a step planner which runs at a frequency of 1000 HZ. The step planner using a simple model is embedded into the inverse-dynamics–based whole body controller (Herzog et al., 2015; Kim et al., 2019) which tackles the inconsistency between the simple model used in high-level planning and full robot dynamics. With the whole body controller, the feet of the robot show great compliance, and this helps the robot to transit between different terrains without any information about the terrain. Due to the fast execution frequency and compliance of the foot, our proposed controller enables the robot to blindly walk over various kinds of moderately uneven terrains including slopes, wave fields, and stairs. Our controller can also handle disturbances from all direction while walking on uneven terrains. We validated our controller on the straight-legged bipedal robot SLIDER (Wang et al., 2020) in Gazebo simulation.

**FIGURE 1:**
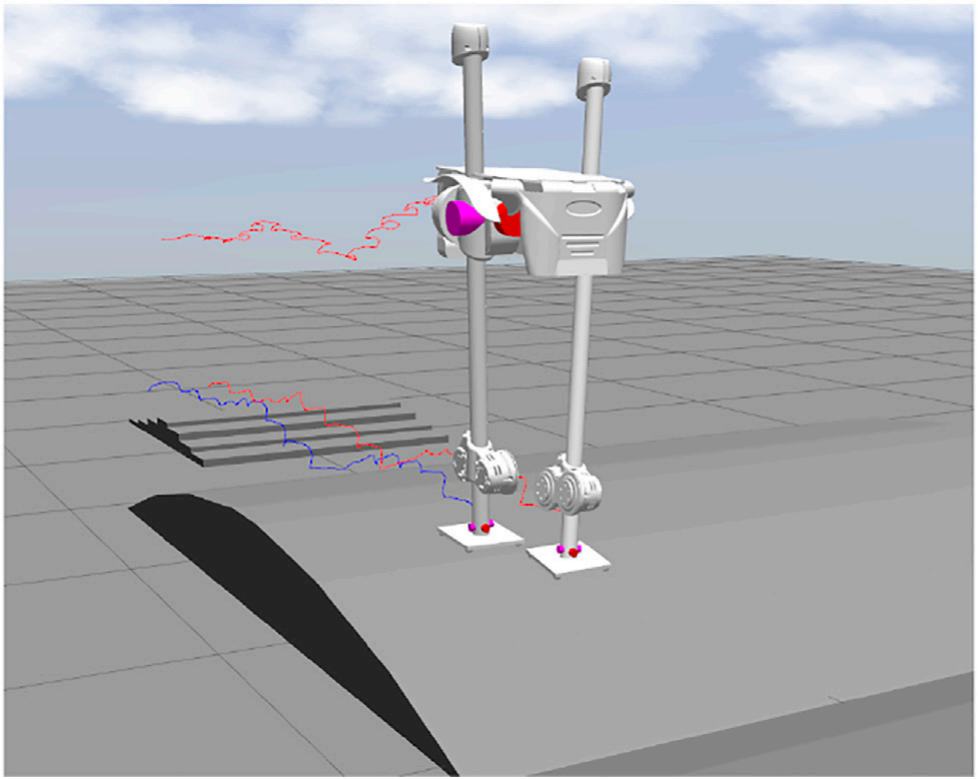
SLIDER robot walks on the uneven terrain. The three traces of trajectories are CoM, left foot, and right foot.

The main contribution of the article is the fast online optimization algorithm which enables bipedal robots to blindly walk over various uneven terrains while resisting disturbances, as shown in Figure 1. With reasonable assumptions, we can decouple the nonlinear 3D aSLIP dynamics and optimize the footstep location and CoM height separately. By proper reformulation, the control of the vertical dynamics can be formulated as QP, and therefore a solution is guaranteed to be found. Our controller is simple to implement and computationally efficient; the optimization of both vertical and horizontal motions can be solved by QP and executed at 1 kHz. A video showing the SLIDER robot walking with the proposed controller in simulation is available at: https://youtu.be/ROyV-ZP8dxA.

## 2 System Overview

### 2.1 The SLIDER Robot

**FIGURE 2:**
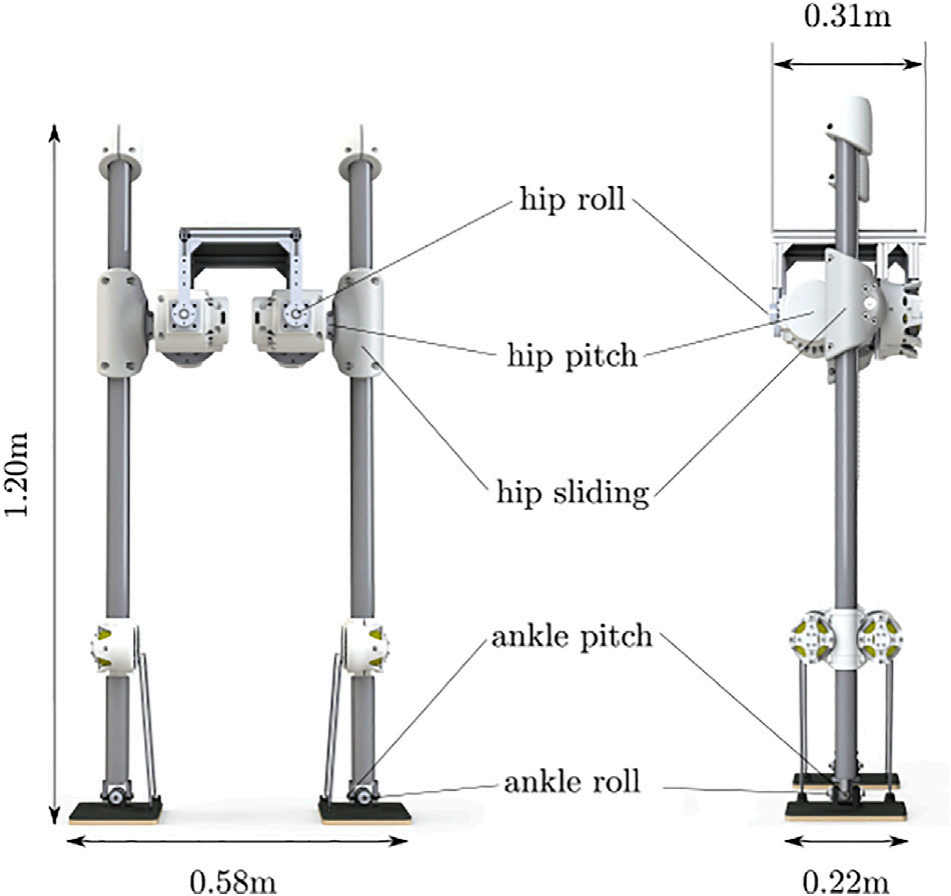
Dimension and joint configuration of the SLIDER robot.

SLIDER is a knee-less bipedal robot designed by the Robot Intelligence Lab at Imperial College London, as shown in Figure 2. SLIDER is 1.2 m tall and has 10 degrees of freedom (DoF), namely hip pitch, hip roll, hip slide, ankle roll, and ankle pitch on each leg. The robot is very lightweight (14.5 kg in total), and most of its weight is concentrated at the pelvis. The legs are made of carbon fiber–reinforced polymer, and each leg weights only 0.4 kg. The prismatic knee joint design is a unique feature of this robot that differentiates it from many other robots with the anthropomorphic design. Due to its sliding mechanism and lightweight leg design, SLIDER can be well approximated with an aSLIP model which can greatly simplify the planning and control problem. Moreover, the lightweight leg with a large range of motion makes the robot suitable for agile locomotion.

### 2.2 The Control Hierarchy

**FIGURE 3:**
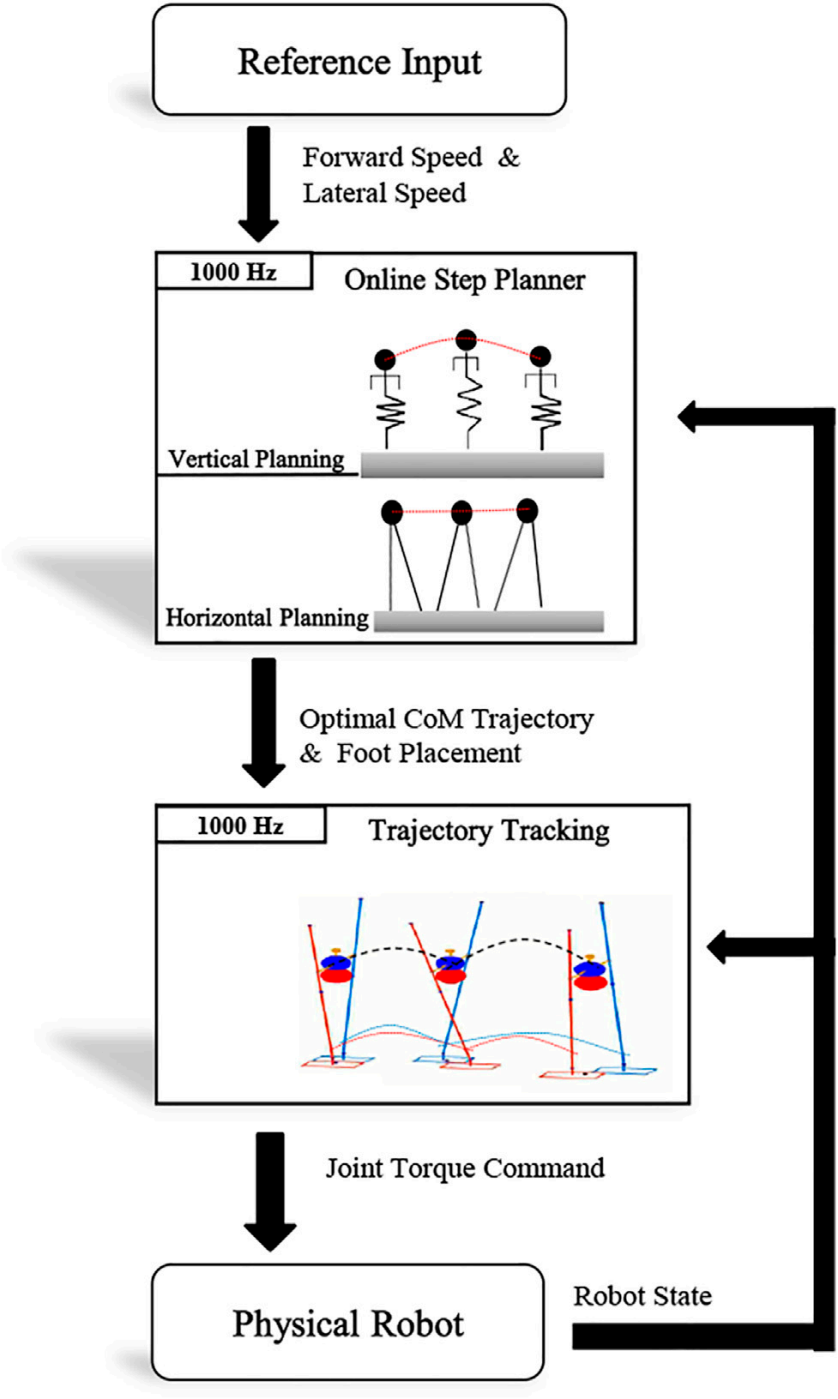
Hierarchical controller with the execution frequency of each level. The high-level trajectory planner takes the desired velocity as input and generates the optimal CoM trajectory along with the foot placement. The low-level whole body controller (Feng et al., 2015; Herzog et al., 2015) considers full dynamics of the robot and tracks the trajectory at a frequency of 1 kHz.

The controller presented has a hierarchical structure as shown in Figure 3. Due to the computational complexity of using full body motion planning, a high-level planner optimizes only the foot placement in *x*, *y* directions and the CoM height using the decoupled aSLIP model online. The low-level whole body controller (Feng et al., 2015; Herzog et al., 2015) tracks the trajectory generated by the high-level planner. The whole body controller considers the full dynamics of the robot and generates the consistent torque command for each joint.

## 3 Online Footstep Planning

Motion planning with the full dynamics of the robot is too computationally expensive to be executed at a fast frequency. Instead, we use reduced order models for online footstep planning: the LIP model is used to plan footsteps in the horizontal plane, and the 1D-actuated spring model is used to generate in the vertical direction.

### 3.1 The Decoupled aSLIP Model

**FIGURE 4:**
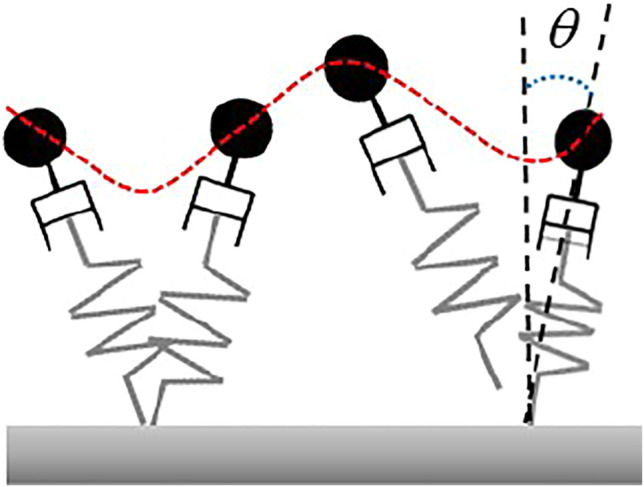
aSLIP model. This model combines the SLIP model with a virtual linear actuator. *θ* is the angle between the inverted pendulum and the vertical axis.

The aSLIP model is different from the classical SLIP model in which the aSLIP model is a combination of the SLIP model and a virtual linear actuator, as shown in Figure 4. Introducing such a virtual actuator gives the aSLIP model the capability to actively modify the reference spring length during each walking phase and makes aSLIP model better at handling vertical changes than the SLIP model.

However, similar to the SLIP model, the dynamics of the aSLIP model presented previously is nonlinear because of the coupling in vertical and horizontal dynamics, and the exact dynamic equation needs to be numerically integrated. An approximate model, which decouples the CoM, states into horizontal and vertical directions will be used in this article. This decoupled approximate model simplifies the nonlinear dynamics of the original aSLIP model and makes the fast online optimization of footstep locations and CoM height possible. To make the approximation valid, two assumptions are made. The first assumption is that the vertical deviation of CoM over each walking period is small relative to the height of the CoM, such that individual steps can be modeled using the LIP model. The second assumption is that the angle the inverted pendulum makes with the vertical axis is small, such that the spring contributes to the CoM’s vertical behavior only. This is reasonable, considering a typical small step length is small compared to the height of the CoM. These assumptions are summarized in Figure 4.

### 3.2 Vertical Dynamics of the Decoupled aSLIP Model

**FIGURE 5:**
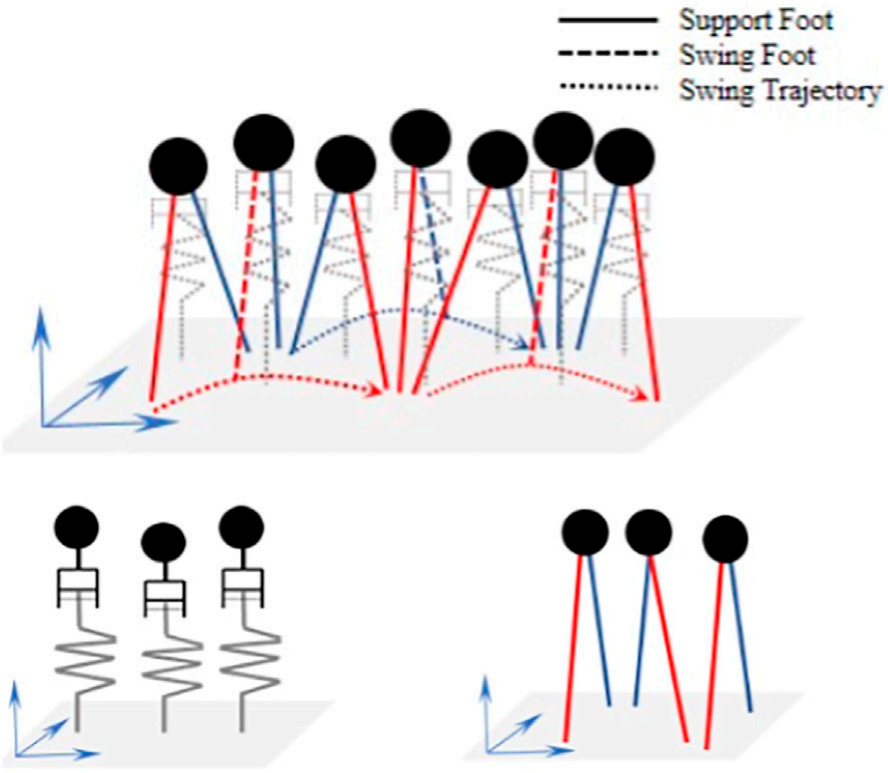
Decoupled aSLIP model during walking (top), with vertical dynamics (bottom left), and horizontal dynamics (bottom right).

The original dynamics equation of the SLIP model is

$$\left[\begin{array}{c} m\ddot{l}\\ ml^{2}\ddot{\theta } \end{array}\right]=\left[\begin{array}{c} ml{\dot{\theta }}^{2}-k\left(l-l_{0}\right)-mg\cos\theta \\ -2ml\dot{l}\dot{\theta }+mgl\sin\theta \end{array}\right],$$ (1)

where *l* is the pendulum length and *θ* is the angle between the pendulum and the vertical axis, as demonstrated in Figure 4. Under the assumption that *θ* is negligible and *l* can be well approximated by the CoM height *z*, the dynamics can be simplified into the decoupled aSLIP model in vertical direction by Eq. 2, as shown in Figure 5, where *r* denotes the reference spring length, and *k* is the stiffness of the spring.

$$m\ddot{z}=-mg+k\left(z-r\right).$$ (2)

Compared with the classical SLIP model, the reference spring length *r*(*t*) is treated as a time-varying optimization variable, which is contributed by the virtual linear actuator. We also constrain *r*(*t*) to change linearly between initial and final values over the phase to facilitate fast computation. Therefore, the reference spring length should satisfy the condition below,

$$r\left(t\right)=r_{0}+\frac{t}{T}\left(r_{T}-r_{0}\right),$$ (3)

where *r*_0_ and *r*_*T*_ represent the reference spring length at the start and at the end of one step, respectively, and *T* is the step duration. We can formulate the dynamic Eq. 2 into a state space equation as,

$$\dot{Z}=\left[\begin{array}{cc} 0 & 1\\ -\omega_{z}^{2} & 0 \end{array}\right]Z+\left[\begin{array}{c} 0\\ \omega_{z}^{2} \end{array}\right]\left(r-\frac{g}{\omega_{z}^{2}}\right),$$ (4)

where the states of CoM in vertical direction are defined as $Z={\left[z,\dot{z}\right]}^{T}$ , and we define $\omega_{z}=\sqrt{\frac{k}{m}}$ . Further, we can denote $r-\frac{g}{\omega_{z}^{2}}$ as *u*_*z*_, and Eq. 5 can be written into a linear state space equation as,

$$\dot{Z}=\left[\begin{array}{cc} 0 & 1\\ -\omega_{z}^{2} & 0 \end{array}\right]Z+\left[\begin{array}{c} 0\\ \omega_{z}^{2} \end{array}\right]u_{z}.$$ (5)

We discretize Eq. 5 with sampling time *T*_*s*_ and can obtain,

$$Z_{k+1}=\Phi \left(T_{s}\right)Z_{k}+\int_{0}^{T_{s}}\Phi \left(T_{s}\right)dt\left[\begin{array}{c} 0\\ \omega_{z}^{2} \end{array}\right]u_{z,k},$$ (6)

where

$$\Phi \left(T_{s}\right)=\left[\begin{array}{cc} \cos\left(\omega_{z}T_{s}\right) & \sin\left(\omega_{z}T_{s}\right)/\omega \\ \omega_{z}\cdot \sin\left(\omega_{z}T_{s}\right) & \cos\left(\omega_{z}T_{s}\right) \end{array}\right];$$ (7)

$$\int_{0}^{T_{s}}\Phi \left(T_{s}\right)dt\left[\begin{array}{c} 0\\ \omega_{z}^{2} \end{array}\right]=\left[\begin{array}{c} 1-\cos\left(\omega_{z}T_{s}\right)\\ \omega_{z}\cdot \sin\left(\omega_{z}T_{s}\right) \end{array}\right].$$

We can also write this equation as:

$$Z_{k+1}=A_{z}\left(T_{s}\right)Z_{k}+B_{z}\left(T_{s}\right)u_{z,k},$$

where *Z*_*k*_ and *Z*_*k*+1_ are CoM states in the vertical direction at time k and k+1, respectively, *A*_*z*_ (*T*_*s*_) and *B*_*z*_ (*T*_*s*_) are time-varying matrices, and *u*_*z*,*k*_ is the control input for the spring at time k. Therefore, *N* future steps can be computed with the given fixed sampling time *T*_*s*_:

$$\begin{array}{c} Z_{1}=A_{z}\left(T_{s}\right)Z_{0}+B_{z}\left(T_{s}\right)u_{z,0}\\ Z_{2}=A_{z}\left(T_{s}\right)Z_{1}+B_{z}\left(T_{s}\right)u_{z,1}\\ \ldots ,\\ Z_{N}=A_{z}\left(T_{s}\right)Z_{N-1}+B_{z}\left(T_{s}\right)u_{z,N-1} \end{array}$$ (8)

where *N* is the number of steps to be optimized which should be more than 1. In this formulation, the control input vector for the spring $\bar{U}=\left[u_{z,0},u_{z,1},\ldots ,u_{z,N-1}\right]$ is the optimization variable. Eq. 8 can also be written in a matrix form for MPC:

$$\bar{Z}=\left[\begin{array}{c} A_{z}\\ A_{z}^{2}\\ \ldots ,\\ A_{z}^{N} \end{array}\right]Z_{0}+\left[\begin{array}{cccc} B_{z} & 0 & 0 & 0\\ A_{z}B_{z} & B_{z} & 0 & 0\\ \ldots & \ldots & \ldots & \ldots ,\\ A_{z}^{N-1}B_{z} & A_{z}^{N-2}B_{z} & \ldots & B_{z} \end{array}\right]{\bar{U}}_{z},$$ (9)

where $\bar{Z}$ and ${\bar{U}}_{z}$ are the vectors of states and control inputs in z direction, respectively.

### 3.3 Horizontal Dynamics of the Decoupled aSLIP Model

Under the assumption that the leg angle *θ* is relatively small and therefore the horizontal and the vertical dynamics can be decoupled, the horizontal dynamics then becomes a classical linear inverted pendulum (LIP) model as shown in Eq. 10, where *z*_0_ is the pendulum height and remains constant within each step but may change between steps. Please note that *z*_0_ is different from the CoM height *z*. Similar to Xin et al., (2019), given the continuous dynamics of LIP model in the *x* direction:

$$\ddot{x}=\frac{g}{z_{0}}\left(x-p_{x}\right).$$ (10)

The discrete state space equation can be reformulated into a linear state space as follows, with the state defined as $X={\left[x,\dot{x}\right]}^{T}$ , and input *u*_*x*_ defined as the footstep positions in *x* direction:

$$X_{k+1}=A_{x}\left(T_{s}\right)X_{k}+B_{x}\left(T_{s}\right)u_{x,k},$$ (11)

where

$$A_{x}\left(T_{s}\right)=\left[\begin{array}{cc} \cosh\left(\omega_{x}T_{s}\right) & \sinh\left(\omega_{x}T_{s}\right)/\omega_{x}\\ \omega_{x}\cdot \sinh\left(\omega_{x}T_{s}\right) & \cosh\left(\omega_{x}T_{s}\right) \end{array}\right];$$

$$B_{x}\left(T_{s}\right)=\left[\begin{array}{c} 1-\cosh\left(\omega_{x}T_{s}\right)\\ \omega_{x}\cdot \sinh\left(\omega_{x}T_{s}\right) \end{array}\right].$$

Similarly, states in the predicted time horizon can also be obtained by:

$$\bar{X}=\left[\begin{array}{c} A_{x}\\ A_{x}^{2}\\ \ldots \\ A_{x}^{N} \end{array}\right]X_{0}+\left[\begin{array}{cccc} B_{x} & 0 & 0 & 0\\ A_{x}B_{x} & B_{x} & 0 & 0\\ \ldots & \ldots & \ldots & \ldots \\ A_{x}^{N-1}B_{x} & A_{x}^{N-2}B_{x} & \ldots & B_{x} \end{array}\right]{\bar{U}}_{x},$$ (12)

where $\bar{X}$ and ${\bar{U}}_{x}$ are the vectors of states and control inputs in *x* direction, respectively. The dynamics in the *y* direction has identical formulation to the dynamics in the *x* direction.

### 3.4 Foot Placement and CoM Trajectory Optimization

The footstep planning can be formulated as a QP problem due to the fact that both horizontal and vertical dynamics are linear and the constraints are linear. The formulation is:

$$\underset{u_{x},u_{y},u_{z}}{\min}\Gamma \;\;\left(\mathrm{cost}\mathrm{function}\right);$$

$$s.t.\;X_{k+1}=A\left(T_{s}\right)X_{k}+B\left(T_{s}\right)u_{k}\;\;\left(\mathrm{dynamcs}\right);$$

$$h\left(p_{j}\right)<0\;\;\left(\mathrm{reachability}\right),$$

where Γ = Γ_1_ + Γ_2_ + Γ_3_ is the cost function term, *X* and *u* are general representations of states and control inputs, respectively, and **p**_*j*_ is the left or right foot position.

The cost formulation is composed of three parts: Γ_1_, Γ_2_, and Γ_3_. The first part is minimizing the difference between the predicted state and the referenced state, which drives the CoM state to reach the desired one from the given current state. The formulation is:

$$\Gamma_{1}=||X_{N}-X_{N}^{ref}|{|}_{P}^{2}+\overset{N-1}{\underset{k=0}{\sum }}||X_{k}-X_{k}^{ref}|{|}_{Q}^{2},$$ (13)

where *P* and *Q* are the weight matrices. We only care about the velocity of CoM in all directions because we want a reactive footstep planner which is not constrained by an absolute reference trajectory. From another point of view, tracking the reference velocity is equivalent to tracking the relative CoM reference position. *X* ^*ref*^ is easy to define with the desired sagittal and frontal velocity. However, the reference velocity in the vertical direction is not constant during one step. To get the reference velocity, we can integrate Eq. 2 and get the continuous dynamics equation:

$$z\left(t\right)=d_{1}\cos\omega_{z}t+d_{2}\sin\omega_{z}t+r\left(t\right)-g/{\omega_{z}}^{2},$$ (14)

where

$$d_{1}=z_{0}-r_{0}+g/{\omega_{z}}^{2};$$ (15)

$$d_{2}={\dot{z}}_{0}/{\omega_{z}}^{2}-\left(r\left(t\right)-r_{0}\right)/\left(T\omega \right).$$ (16)

In this equation, *z*_0_ and ${\dot{z}}_{0}$ are the CoM vertical position and velocity at the start of the current step, respectively. Then, the time-varying reference velocity in z direction can be derived from the continuous dynamics.

The second part is minimizing the difference between the reference control input $u_{k}^{ref}$ and the optimized control input *u*_*k*_ and R is the weight matrix.

$$\Gamma_{2}=\overset{N-1}{\underset{k=0}{\sum }}||u_{k}-u_{k}^{ref}|{|}_{R}^{2}.$$ (17)

For the reference control input in z direction, recall that the spring length is constrained to change linearly, as indicated by Eq. 3. If we denote Δ*r*_*i*_ as the change of spring length during one sampling time *T*_*s*_ at step *i*, then it is clear that Δ*r*_*i*_ is constant throughout step *i*, and the reference control input in z direction ${\bar{U}}_{z}^{ref}$ is therefore obtained as below:

$${\bar{U}}_{z}^{ref}=\left[\begin{array}{c} r_{0}I_{N_{r}}\\ 0_{N_{s}}\\ \ldots \\ 0_{N_{s}} \end{array}\right]+\left[\begin{array}{cccc} 1 & 0 & \ldots & 0\\ 1 & 1 & \ldots & 0\\ 1 & 1 & \ldots & 0\\ 1 & 1 & \ldots & 1 \end{array}\right]\left[\begin{array}{c} 0_{N_{r}}\\ \Delta r_{1}I_{N_{s}}\\ \ldots \\ \Delta r_{N_{steps}}I_{N_{s}} \end{array}\right],$$ (18)

where *N*_*r*_ is the remaining number of sampling points for the current step and *N*_*s*_ is the number of sampling points for one step. *N*_*steps*_ is the number of predicted steps.

For the horizontal trajectory planning, the input of the system is the footstep position. The reference control input in *x* direction ${\bar{U}}_{x}^{ref}$ is then obtained according to the current support foot position *P*_0_, and the difference between two consecutive steps Δ*P*_*x*,*i*_.

$${\bar{U}}_{x}^{ref}=\left[\begin{array}{c} I_{N_{r}}\\ 0_{N_{s}}\\ \ldots ,\\ 0_{N_{s}} \end{array}\right]P_{x,0}+\left[\begin{array}{ccc} 0_{N_{r}} & \ldots & 0_{N_{r}}\\ I_{N_{s}} & \ldots & 0_{N_{s}}\\ \ldots & \ldots & \ldots ,\\ 0_{N_{s}} & \ldots & I_{N_{s}} \end{array}\right]\left[\begin{array}{c} P_{x,1}\\ P_{x,2}\\ \ldots ,\\ P_{x,N_{steps}} \end{array}\right];$$ (19)

$$\left[\begin{array}{c} P_{x,1}\\ P_{x,2}\\ \ldots ,\\ P_{x,N_{step}} \end{array}\right]=\left[\begin{array}{c} I_{N_{r}}\\ I_{N_{s}}\\ \ldots ,\\ I_{N_{s}} \end{array}\right]P_{x,0}+\left[\begin{array}{cccc} 1 & 0 & 0 & 0\\ 1 & 1 & 0 & 0\\ \ldots & \ldots & \ldots & \ldots ,\\ 1 & 1 & \ldots & 1 \end{array}\right]\left[\begin{array}{c} \Delta P_{x,1}\\ \Delta P_{x,2}\\ \ldots ,\\ \Delta P_{x,N_{step}} \end{array}\right].$$

The third part is tracking the desired change of the control input between two consecutive steps, with *d*_*i*_ denoting the desired difference and *W* denoting weights. For the vertical trajectory planning, we set the desired difference equal to zero; this means the footstep planner tries to keep a constant CoM height during one step to make the decoupled aSLIP assumption valid.

$$\Gamma_{3}^{z}=\overset{N_{steps}}{\underset{i=1}{\sum }}||\Delta r_{i}-d_{i}^{z}|{|}_{W}^{2}=\overset{N_{steps}}{\underset{i=1}{\sum }}||\Delta r_{i}|{|}_{W}^{2}.$$ (20)

For horizontal trajectory planning,

$$\Gamma_{3}^{x,y}=\overset{N_{steps}}{\underset{i=1}{\sum }}||\Delta P_{i}^{x,y}-d_{i}^{x,y}|{|}_{W}^{2}.$$ (21)

In *x* direction, $d_{i}^{x}$ is the step length which is calculated by the desired speed multiplied by the step time. In *y* direction, $d_{i}^{y}$ is defined as $d_{i}^{y}=d_{step}*2*{\left(-1\right)}^{j}$ , where *d*_*step*_ is the desired inter-feet clearance distance, and *j* is the flag for the supporting foot, 0 stands for left support, and 1 stands for right support. This relative distance regularization term is introduced to keep the feet away from each other to avoid self-collision. Moreover, as this part of the cost function only includes relative distances, it helps to produce a reactive footstep planner which keeps the robot walking even when unexpected disturbance is applied.

The reachability constraint is responsible for making sure the footstep location is physically possible,

$$L-r^{x,y}<p_{j}-c<L+r^{x,y},$$ (22)

where **L** is the nominal offset of the foot position from the CoM of the robot, **r** ^*x*,*y*^ is the reachability constraint in the *x* and *y* directions, and **c** is the CoM position.

## 4 Trajectory Tracking

The low-level trajectory tracking generates corresponding torques for each joint to minimize the difference between the actual body trajectory and the desired trajectory given by the high-level trajectory planner. Since the trajectory planning does not include the full body dynamics, the dynamic inconsistency of the torque command generated by the high-level planner is significant. The whole body controller solves the inverse dynamics based on the full robot dynamics.

### 4.1 Rigid Body Dynamics

The walking robot is modeled as a floating-based rigid body system with coordinates *q* = [*q*_*b*_, *q*_*r*_]. Here, $q_{b}\in R^{7}$ represents the position and orientation of the floating base using quaternions, and $q_{r}\in R^{10}$ represents the joint configuration. Inspired by Herzog et al., (2015), the full dynamics can be decomposed into an underactuated part and an actuated part:

$$\left[\begin{array}{c} M_{f}\\ M_{a} \end{array}\right]\ddot{q}+\left[\begin{array}{c} H_{f}\\ H_{a} \end{array}\right]=\left[\begin{array}{c} 0\\ S_{a} \end{array}\right]\tau +\left[\begin{array}{c} J_{f}^{\text{T}}\\ J_{a}^{\text{T}} \end{array}\right]f,$$ (23)

where ***M***, ***H***, ***S***_*a*_, ***τ***, ***J***, and ***f*** are the mass matrix, the Coriolis force vector and the gravitation force vector, the actuator selection matrix, the joint torques vector, the stacked contact Jacobian, and the reaction force vector, respectively. The subscripts *f* and *a* indicate the floating part and the actuated part, respectively.

### 4.2 Contact Force Constraint

Friction is an important contributing factor to the stability of walking to prevent the foot from slipping. We use the pyramidal friction model which is a linear inequality constraint for fast optimization:

$$f_{z}\geq 0,\;|f_{x}|\leq \frac{\mu f_{z}}{\sqrt{2}},\;|f_{y}|\leq \frac{\mu f_{z}}{\sqrt{2}},$$ (24)

where *μ* is the friction coefficient.

### 4.3 Swing Foot Trajectory Generation

The swing foot trajectory is generated using a fifth-order polynomial. The start and final positions and velocities and accelerations are specified, and a parametric quintic curve is generated in *x*, *y*, and *z* directions. The polynomial in *z* direction has two halves, with the predefined foot height and zero velocity at the midpoint. The start and final positions in *z* direction are calculated using the estimated CoM *z* position at the start of current step. Because the robot is walking blindly, we do not define a trajectory for the foot orientation, and this allows the foot to be compliant to a range of surfaces.

### 4.4 Whole Body Controller

The whole-body controller takes the responsibility of computing the joint torques to achieve the desired motions defined in the operational space while respecting a set of constraints. The tasks of interest in this article are the CoM position, the pelvis orientation, the angular momentum of the robot, and the feet positions and orientations. The task for the linear motion can be expressed as:

$$J_{\text{T}}\ddot{q}={\ddot{x}}^{\text{cmd}}-{\dot{J}}_{\text{T}}\dot{q};$$ (25)

$${\ddot{x}}^{\text{cmd}}={\ddot{x}}^{\text{des}}+K_{\text{P}}^{\text{pos}}\left(x^{\text{des}}-x\right)+K_{\text{D}}^{\text{pos}}\left({\dot{x}}^{\text{des}}-\dot{x}\right),$$ (26)

where ***J***_T_ is the translational Jacobian for the task and ***x*** is the actual position of the link. For the task of angular momentum, the centroidal momentum matrix (Orin et al., 2013) is used as the Jacobian for the task. In uneven terrain walking, the angular momentum task is defined as a damping task that damps out excess angular momentum to make the walking robot more stable. For the task of the angular motion, the command can be formulated as:

$$J_{\text{R}}\ddot{q}={\dot{\omega }}^{\text{cmd}}-{\dot{J}}_{\text{R}}\dot{q};$$ (27)

$${\dot{\omega }}^{\text{cmd}}={\dot{\omega }}^{\text{des}}+K_{\text{P}}^{\text{ang}}\left(AngleAxis\left(R^{\text{des}}R^{T}\right)\right)+K_{\text{D}}^{\text{ang}}\left(\omega^{\text{des}}-\omega \right),$$ (28)

where ***J***_R_ is the rotational Jacobian for the task, ***R*** and ***R*** ^des^ denote the actual and desired orientation of the pelvis link, respectively, AngleAxis () maps a rotation matrix to the corresponding axis–angle representation, and $\omega \in R^{3}$ is the angular velocity of the link. The angular motion is needed because when walking on the uneven terrain, the pelvis orientation needs to be regulated around a nominal orientation to maintain a good posture. We set small values for $K_{\text{P}}^{\text{ang}}$ and $K_{\text{D}}^{\text{ang}}$ in the foot orientation task to make the ankle compliant so that the foot can adapt to different terrains. The whole body controller can be formulated as a QP problem as follows:

$$\underset{\ddot{q},\;f}{\min}\;\|A\ddot{q}+\dot{A}\dot{q}-X^{\text{cmd}}{\|}_{W}^{2};$$ (29)

$$s.t.\;M_{f}\ddot{q}-J_{f}^{\text{T}}f=-H_{f}\;\;\left(\mathrm{floatingbasedynamics}\right);$$

$$Pf\leq 0\;\;\;\left(\mathrm{pyramidalfrictioncone}\right);$$

$$S_{a}^{-1}\left(M_{a}\ddot{q}+H_{a}-J_{a}^{\text{T}}f\right)\in \left[\tau_{\min},\;\tau_{\max}\right]\;\;\left(\mathrm{inputlimits}\right),$$

where ***A*** is a stack of the Jacobian matrices for the tasks of interest, ***X*** ^cmd^ is a stack of the commanded accelerations, and ***W*** is the weighting matrix, and ***P*** denotes the linearized friction cone. We treat the unilateral contact constraint as a soft constraint by simply assigning a large weight on the desired zero acceleration of the foot (Kuindersma et al., 2016; Apgar et al., 2018). This can speed up the optimization, and it is reported in Feng et al., (2015) that this gives better stability. The output joint torque commands ***τ*** at each control iteration can be computed by

$$\tau =S_{a}^{-1}\left(M_{a}\ddot{q}+H_{a}-J_{a}^{\text{T}}f\right).$$ (30)

## 5 Results

**FIGURE 6:**
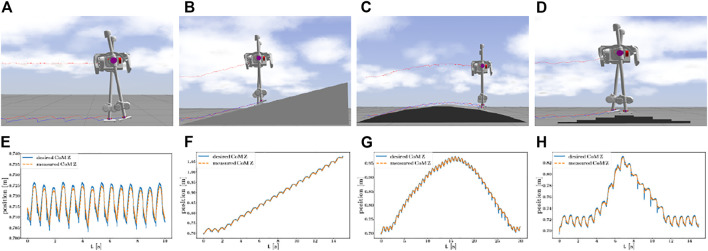
Snapshots and plots of SLIDER blindly walking on different kinds of terrains, with a constant forward velocity of 0.3 m/s (a ∼c) and 0.6 m/s (d). On the top row, from left to right, (a) ∼(d) walk on the flat terrain. Walk on a slope with an angle of 15°. Walk on a wave field approximated by slopes with 3 different angles: 15, 10, and 5°. Walk over stairs; the successive elevation changes are +2 cm, +2 cm, +3 cm, +3 cm, −2 cm, −3 cm, −2 cm, and −3 cm. On the bottom row, from left to right, (e) ∼(f) the plots of desired and measured CoM *z* position in corresponding scenarios.

This section discusses implementation details and simulation results of the SLIDER robot walking on different kinds of uneven terrains including slopes, wave fields, and stairs, as shown in Figure 6. All the experiments are included in the accompanying video https://youtu.be/ROyV-ZP8dxA.

### 5.1 Implementation

Both the high-level footstep planner and low-level trajectory tracking controller are implemented in C++ for real-time performance. In the whole body controller, we use Pinocchio (Carpentier et al., 2015–2021) to compute the full rigid body dynamics and qpOASES (Ferreau et al., 2014) to solve the related QP problem in both levels. All experiments were carried out in the robot simulation environment Gazebo (Koenig and Howard, 2004) with the physics engine ODE (Drumwright et al., 2010), using the full dynamics of the real SLIDER robot. The communication through different levels of the control hierarchy is achieved through ROS.

The decoupled aSLIP parameters are set to match with the physical SLIDER robot, here *r*_0_ = 0.715 m, m = 14.5 kg, and k = 1470 N/m. The step duration is chosen to be 0.7 s, and the foot height in the swing foot trajectory generation is 5 cm. The sampling time in the discrete-time MPC is 0.1 s in *x* and *y* directions and 0.05 s in *z* direction. The motion planner predicts four steps in horizontal states and one step in the vertical state. We use the same parameters among all walking experiments except that the forward velocity is different. In the stair-walking experiment, the robot has to walk faster otherwise the foot might hit the edge of the stair.

### 5.2 Flat Ground Walking

We first validate our approach on the flat ground. Because there are no changes in the *z* direction on the flat ground, the *z* position of CoM oscillates around *r*_0_, as shown in Figures 6A,E.

### 5.3 Walking on the Smooth Uneven Terrain

**FIGURE 7:**
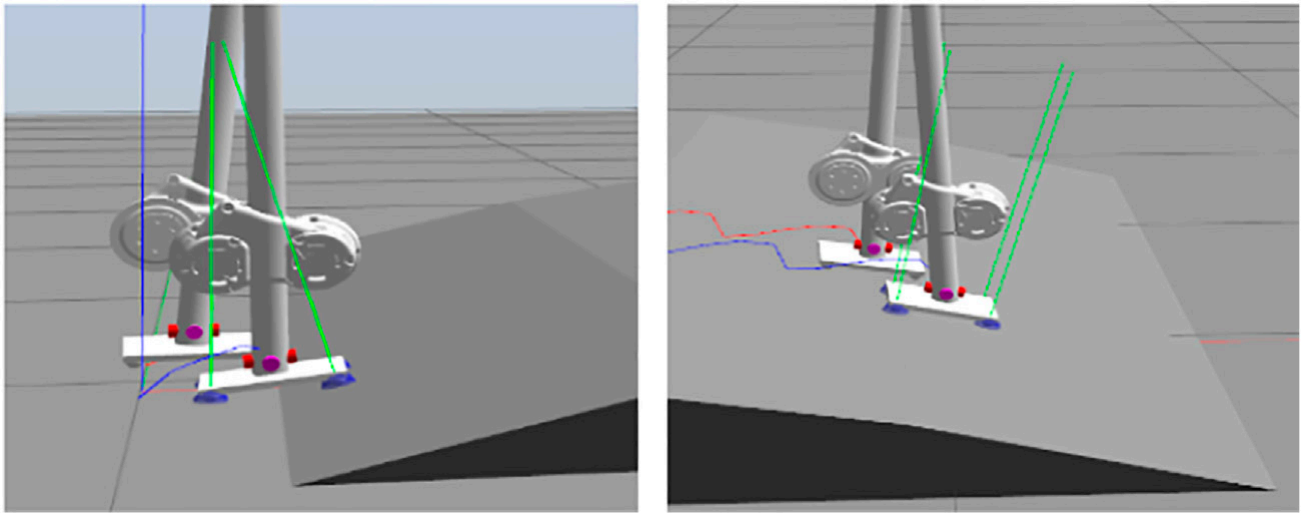
With the whole body controller, the foot is compliant, and the robot can perform transitions to different terrains even though the robot does not know how the terrain looks like. This makes the robot robust to unobserved terrain variations. The green lines indicate the magnitude and direction of the ground reaction forces. The blue and red lines show the trace of the right and left foot centers, respectively.

We then validate our approach on slopes and wave fields where the change of terrain height is smooth. With a forward velocity of 0.3 m/s, the SLIDER robot can walk on a slope with 15° and a wave field approximated by slopes with three different angles: 15, 10, and 5°, as shown in Figures 6B,C,F,G. In the experiment, the whole body controller plays an important role in making the robot remain robust to unobserved terrain variations. By properly tuning the PD gain of the foot orientation task in the whole body controller, the foot is compliant to adapt to large terrain variations, as shown in Figure 7.

**FIGURE 8:**
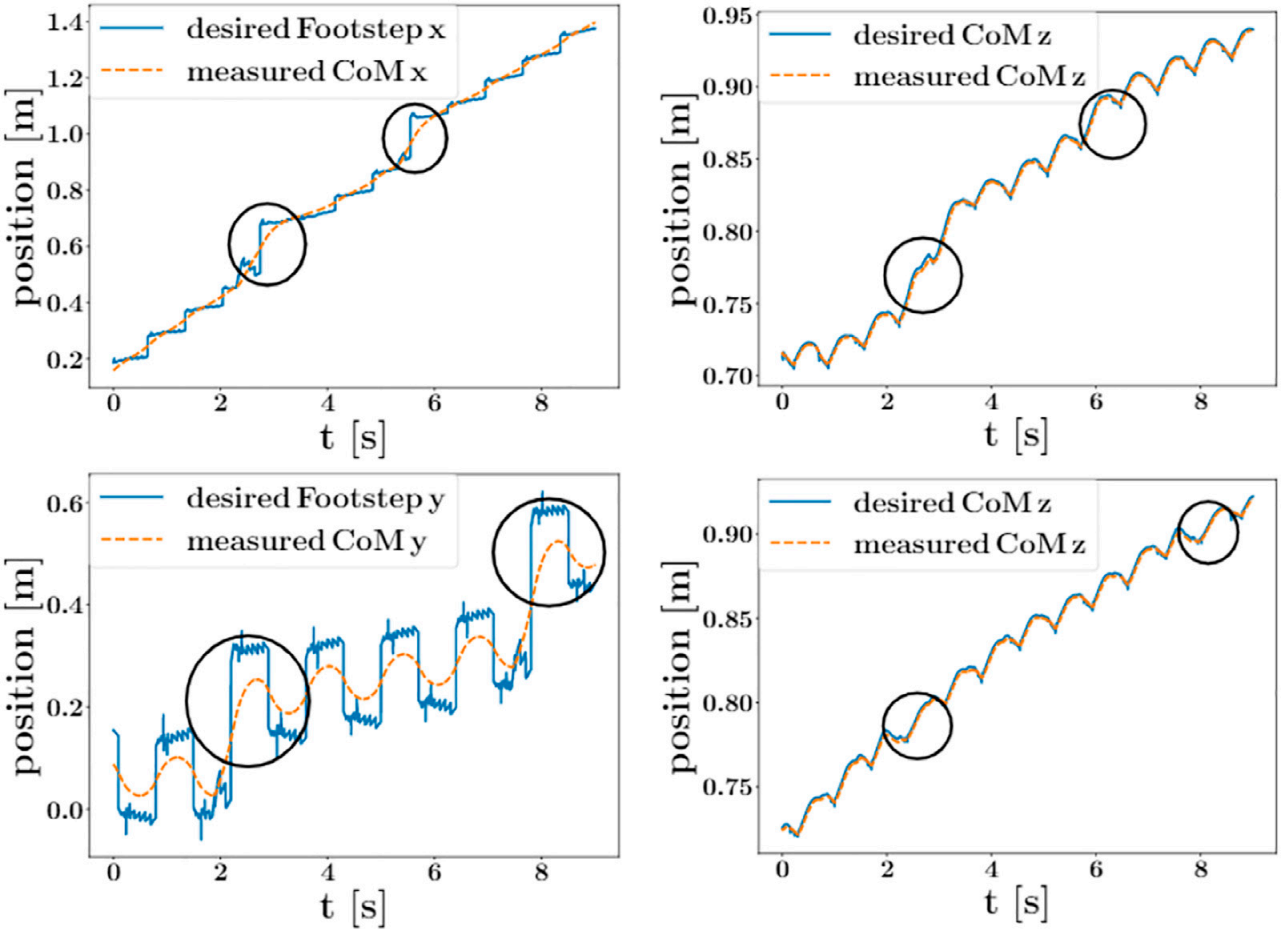
Comparison between the proposed aSLIP planner with the LIP planner when walking on a 15° slope. Top left is the snapshot showing SLIDER walking using a LIP planner; after two steps, the robot stops walking forward because LIP does not take height change into account. Top right is the snapshot showing SLIDER walking using our aSLIP planner where the robot is able to walk forward successfully. Bottom is a plot showing the comparison between the desired CoM height of LIP and aSLIP planners.

Further experiments, such as pushing the robot while it was walking on uneven terrains, were performed. As shown in Figure 8, the robot was pushed in *x* or *y* direction twice while it was walking on a wave field. When the robot was pushed in *x* direction, the robot quickly took a large step in *x* direction to regulate the CoM velocity back to the desired velocity. There is also a big change in *z* direction because the wave field is ascending in *x* direction when the robot got pushed, but the motion quickly got stabilized. In the case of *y* direction push, the robot also took a large step in *y* direction and stabilized in one step. As the terrain height is only changing along *x* direction, there is no big change in *z* direction in this case.

**FIGURE 9:**
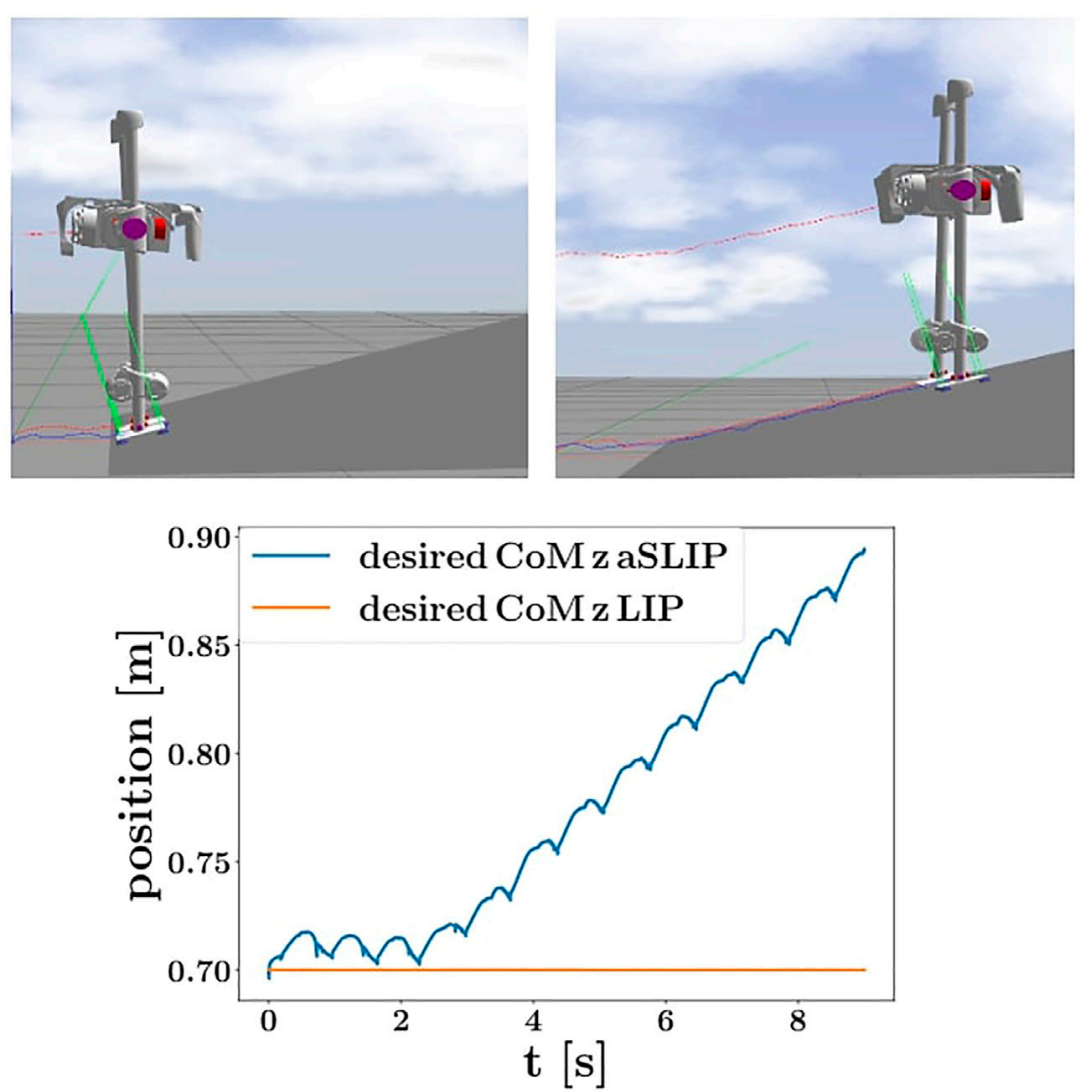
Plots showing the states of CoM when the robot got pushed in *x* or *y* direction while walking on a wave field at a speed of 0.3 m/s. The pushes are indicated with circles. All impulses are applied with a value of 40 N for a duration of 0.1 s. Top row: states of CoM when got pushed in *x* direction. Bottom row: states of CoM when got pushed in *y* direction.

We also compared our proposed planner with a planner using LIP, as shown in Figure 9. The robot with a LIP planner is unable to walk up a 15° slope as the LIP planner assumes a constant CoM height, as shown in the bottom plot in Figure 9. The aSLIP planner enables SLIDER to walk uphill because it adapts to height changes.

### 5.4 Walking on the Discrete Uneven Terrain

The discrete uneven terrain provides bigger instantaneous variations to terrain height than the smooth uneven terrain. As shown in Figure 6D, the robot walked blindly over a set of stairs with a biggest height change of 3 cm. The robot walked with a forward velocity of 0.6 m/s and a foot height of 5 cm in the swing foot phase. As shown in Figure 6H, the CoM Z position exhibits large variations when walking over stairs but was stabilized quickly. In our experiment, the highest step the robot can walk over is 3 cm, for higher steps the foot would hit the edge of the stair and get stuck.

## 6 Discussion

The proposed method is applied to the SLIDER robot and demonstrates successful blind walking on various uneven terrains and the robustness to disturbances due to fast MPC. Because of SLIDER’s lightweight legs, the robot can perform fast leg movements which helps to stabilize the robot. Furthermore, the proposed method is general and can easily be applied to other legged robots.

There are other techniques dealing with variable height CoM trajectories, for example, DCM-based approaches (Englsberger et al., 2013; Caron and Kheddar, 2017). But these techniques require the controller to be terrain-aware to plan future CoM motion. Our proposed controller adjusts the CoM height online within each step by following a spring dynamics. This has two advantages: first, the controller does not require terrain information; second, the spring dynamics enables the vertical compliance of the robot so that the robot is robust to unexpected height variations.

The author observed that the tracking error is larger when the robot walks down the wave field or stairs than walking up, as shown in Figures 6G,H. This happens because the feet are still in the air at the end of one step when the robot walks down. The unexpected sudden drop of the feet at the start of the next step gives the robot a large impact. For robot walking upward, the effect of early touchdown can be alleviated by the compliant feet and vertical compliance of the CoM. To improve, a controller with contact detection or terrain information can achieve a smaller tracking error.

## 7 Conclusion and Future Work

We present an online optimization algorithm, which enables robots to blindly walk over various kinds of uneven terrains while resisting pushes. The high-level motion planner performs fast online optimization on footstep locations and CoM height, and the low-level inverse-dynamics–based whole body controller tracks the trajectory. We show in simulation that using this controller, the robot SLIDER can walk over slopes, wave fields, and stairs without any terrain information and can also recover from pushes while walking. Future work will involve implementing our approach on the real SLIDER robot and also incorporating perception information into the high-level step planner.

## Data Availability

The original contributions presented in the study are included in the article/Supplementary Material; further inquiries can be directed to the corresponding author.
